# Supplementary material for: Distinct Role of γ-Synuclein in the Regulation of Motor Performance and Behavioral Responses in Mice
Source: Biomedicines. 2026 Jan 2;14(1):92. doi: 10.3390/biomedicines14010092 (PMC12839295; doi:10.3390/biomedicines14010092)
Supplement: Supplementary file 1 [file biomedicines-14-00092-s001.zip › FigureS1.pdf]

Figure S1. Uncropped Western blots for synaptic markers in the brains of 13-month-old synuclein KO (γ-KO, αβ-KO, and αβγ-KO) and WT mice.

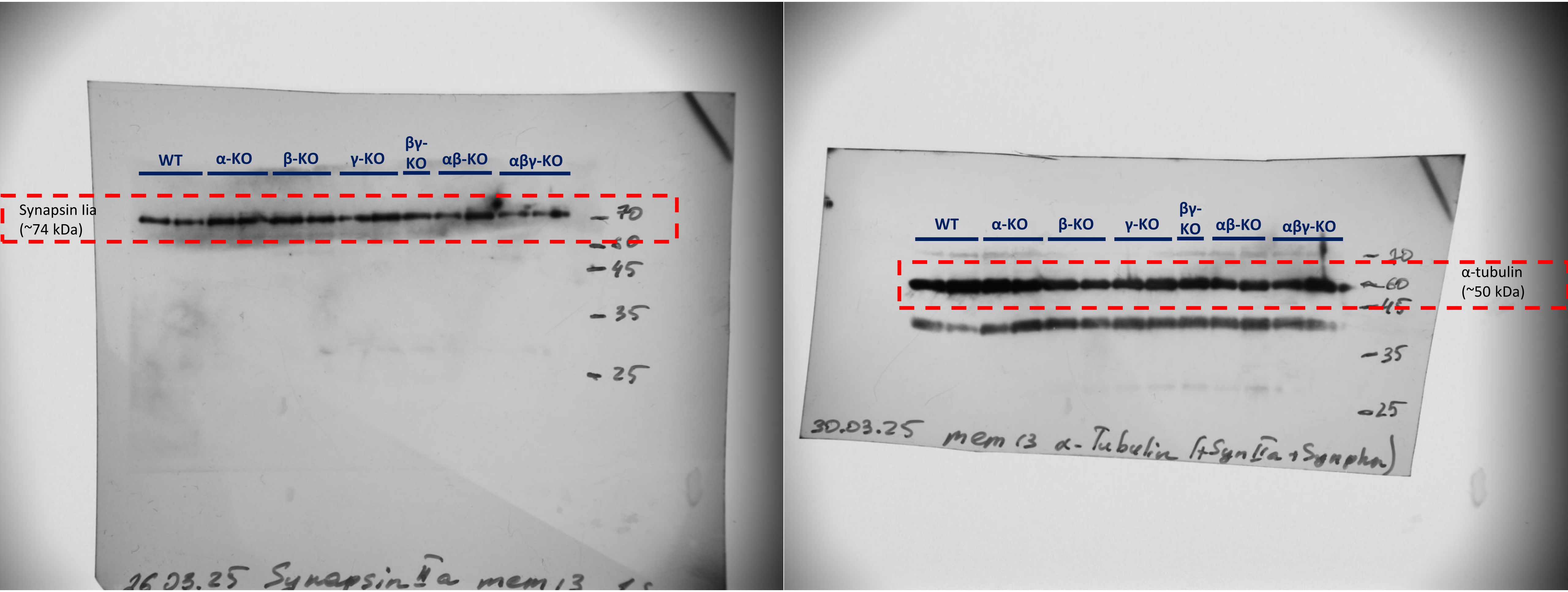

Striatum

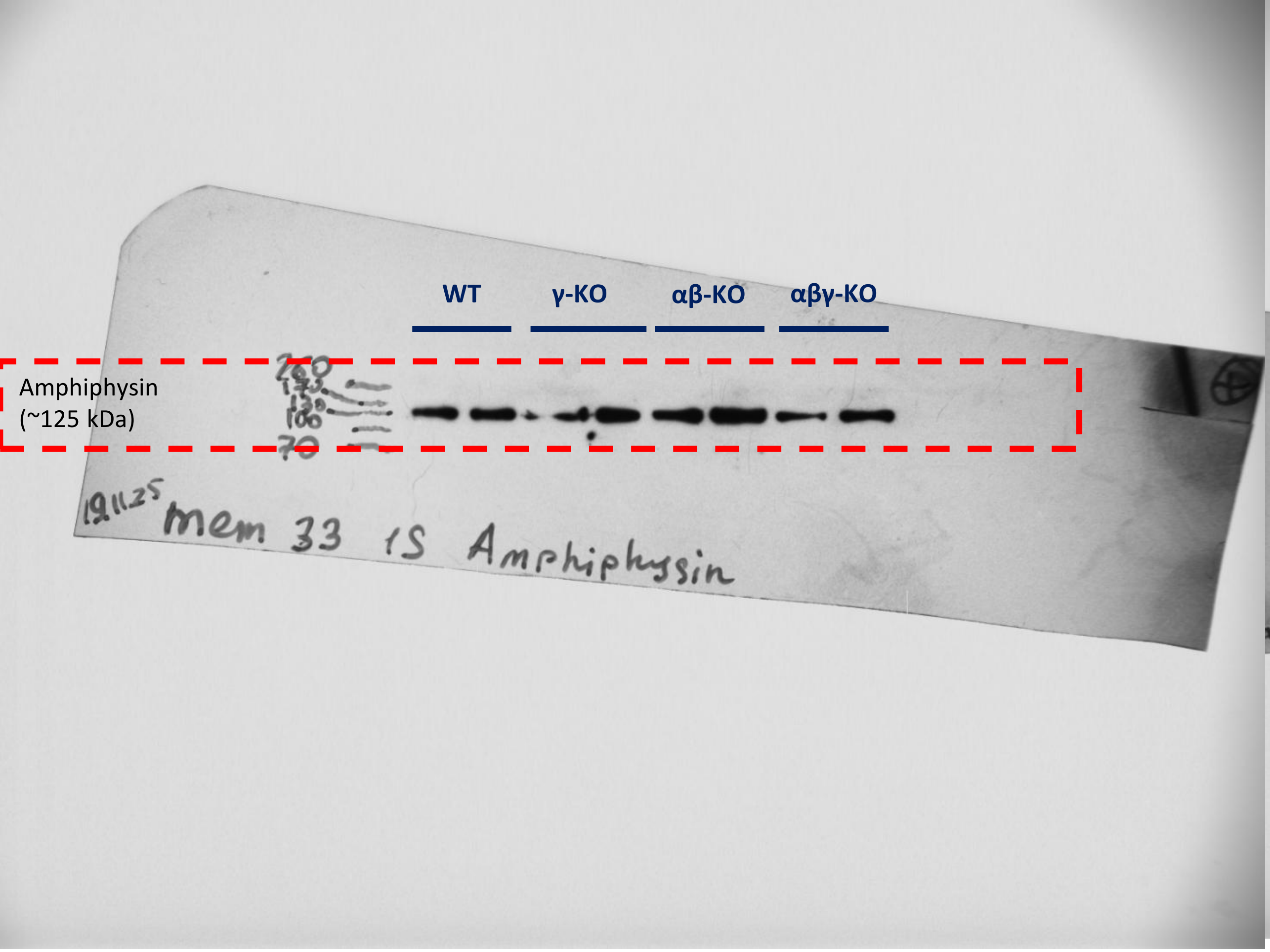
WT
γ-KO
αβ-KO
αβγ-KO
Amphiphysin
(~125 kDa)
mem 33 1S Amphiphysin

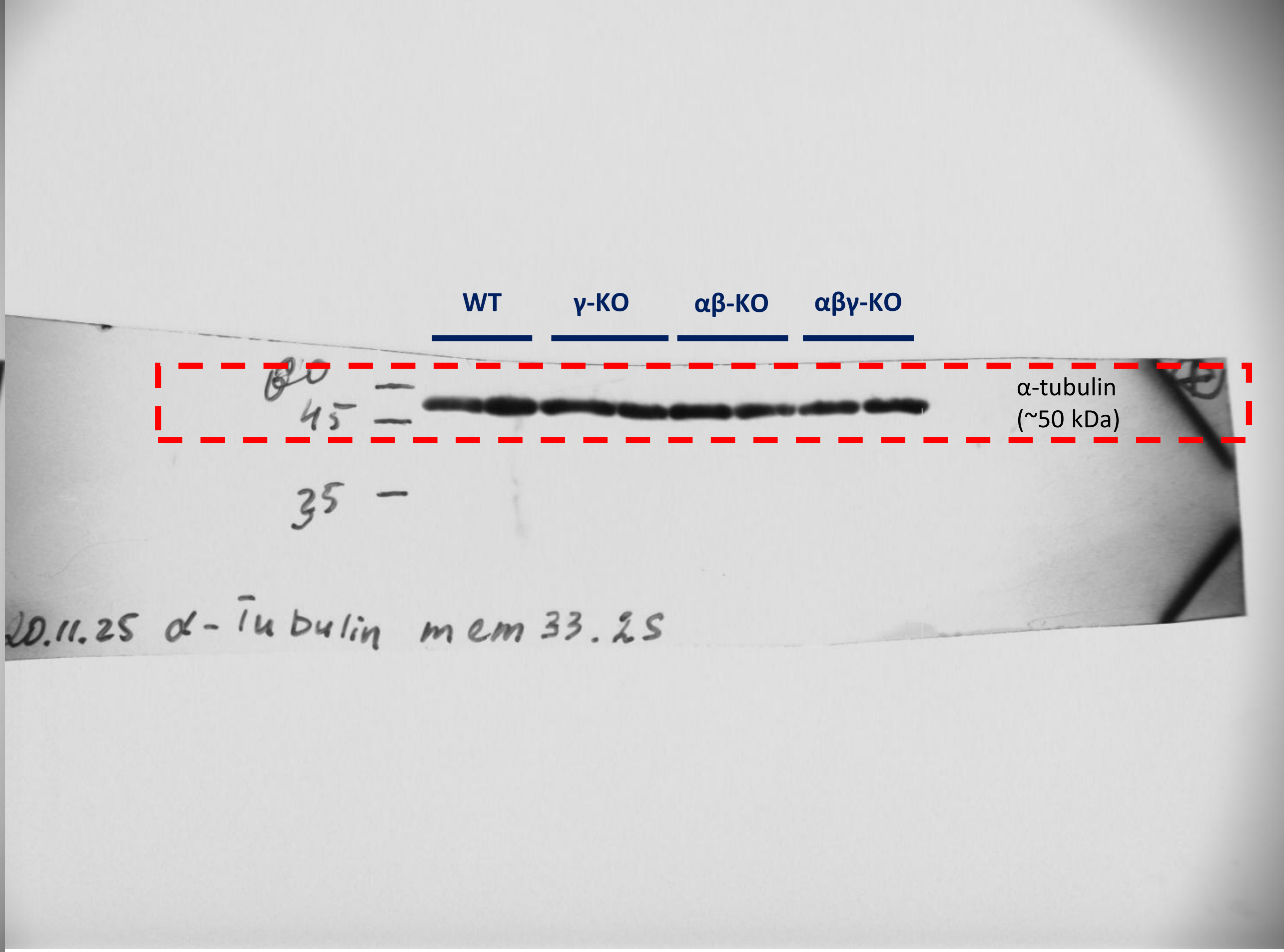
WT
γ-KO
αβ-KO
αβγ-KO
α-tubulin
(~50 kDa)
45
35
20.11.25 α-Tubulin mem 33.2S

Striatum

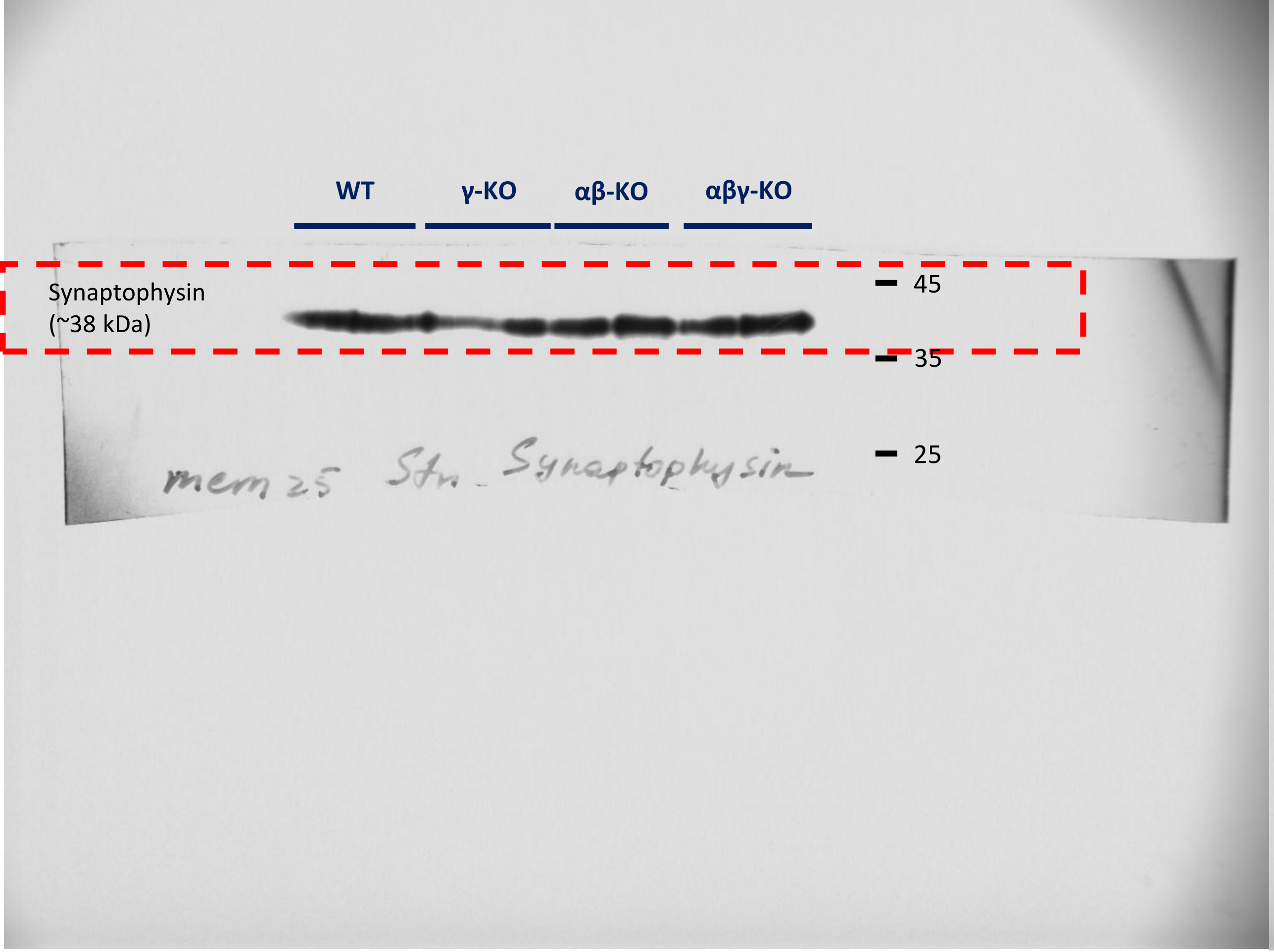
WT
γ-KO
αβ-KO
αβγ-KO
Synaptophysin
(~38 kDa)
45
35
25
mem 25 Str Synaptophysin

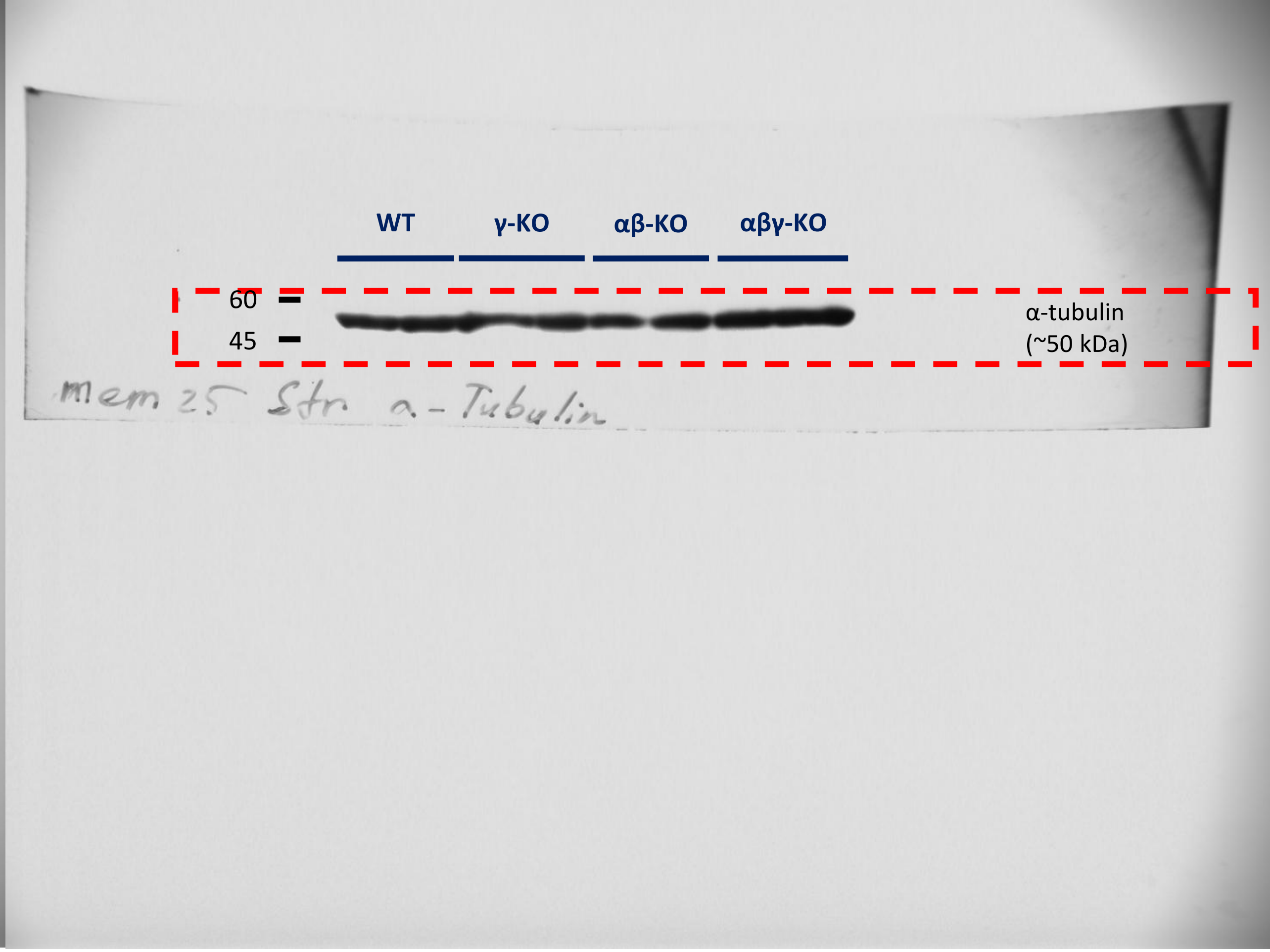
WT
γ-KO
αβ-KO
αβγ-KO
60
45
α-tubulin
(~50 kDa)
mem 25 Str α-Tubulin

Striatum

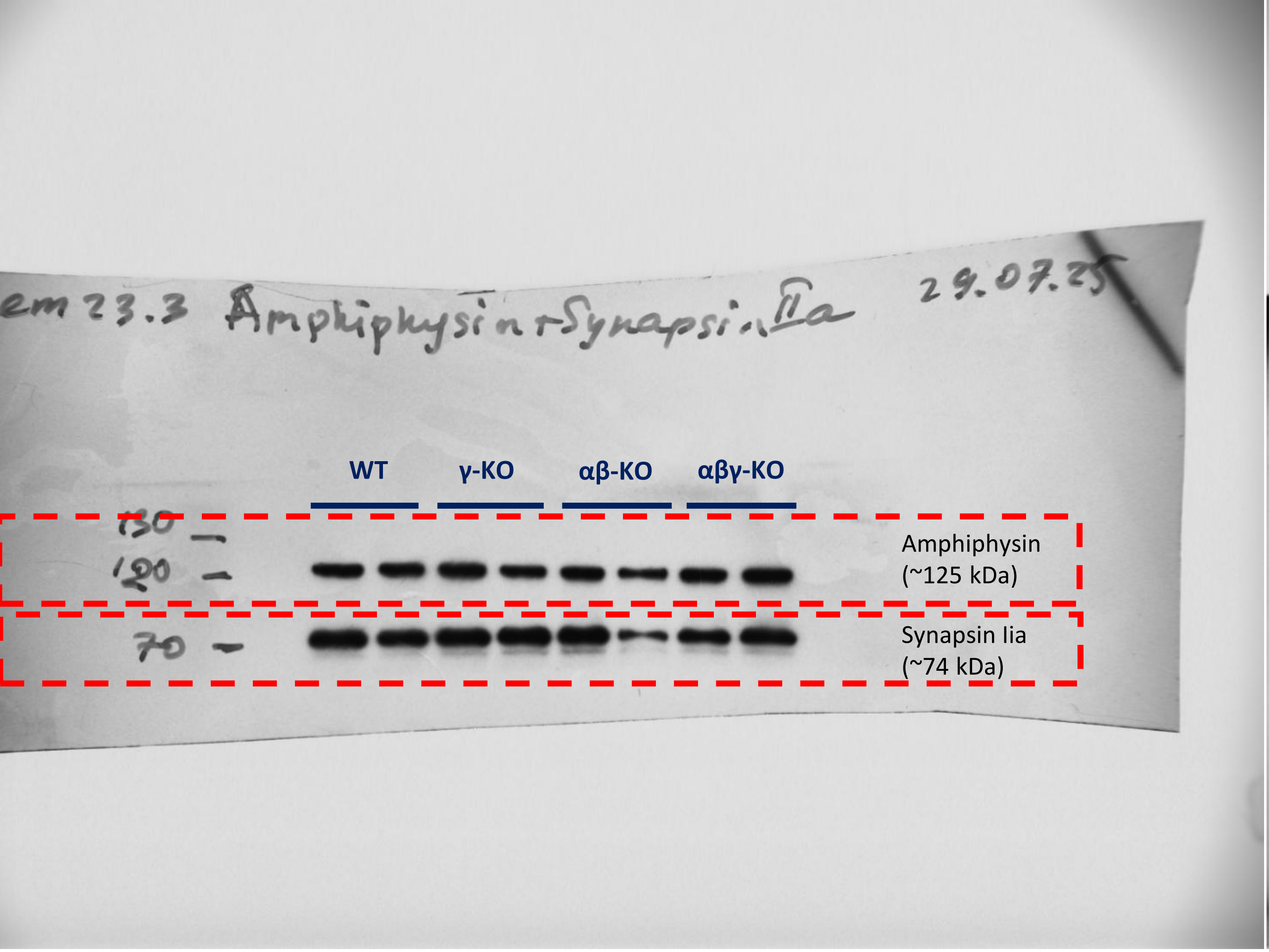
WT
γ-KO
αβ-KO
αβγ-KO
Amphiphysin
(~125 kDa)
Synapsin Iia
(~74 kDa)

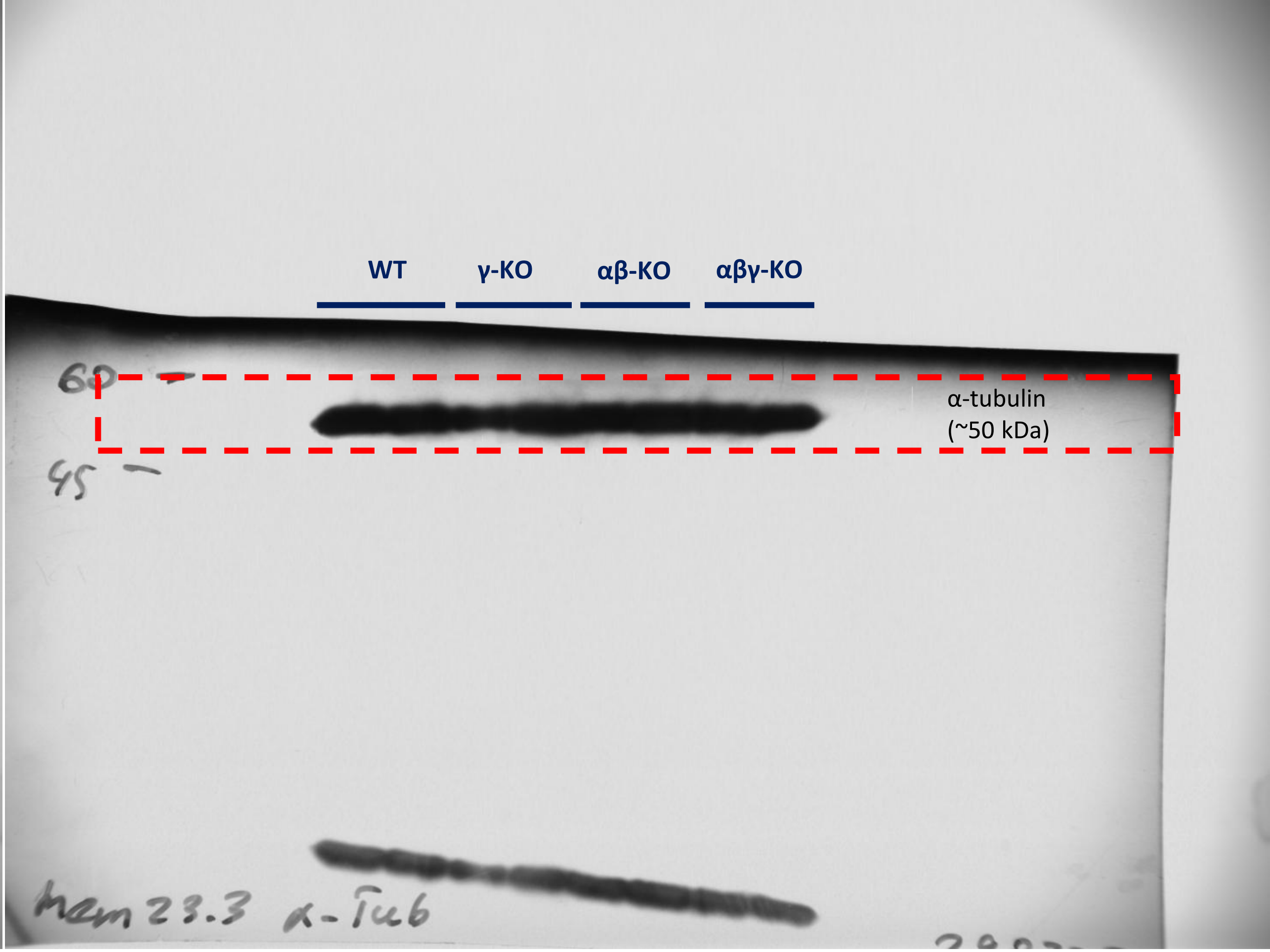
WT
γ-KO
αβ-KO
αβγ-KO
α-tubulin
(~50 kDa)

Cortex

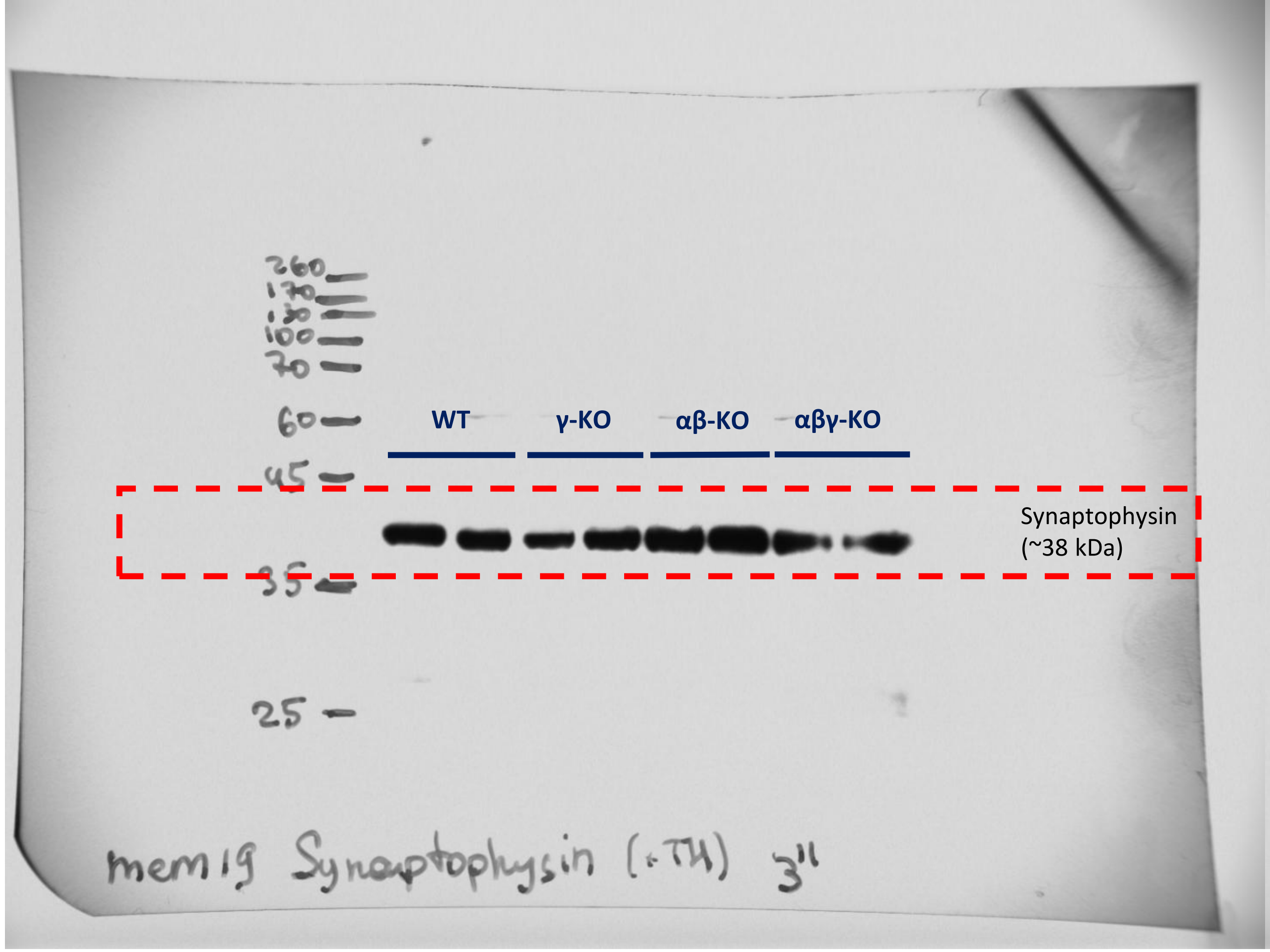
260
170
130
100
70
60
45
35
25
WT
γ-KO
αβ-KO
αβγ-KO
Synaptophysin
(~38 kDa)
mem 1g Synaptophysin (r.TH) 3''

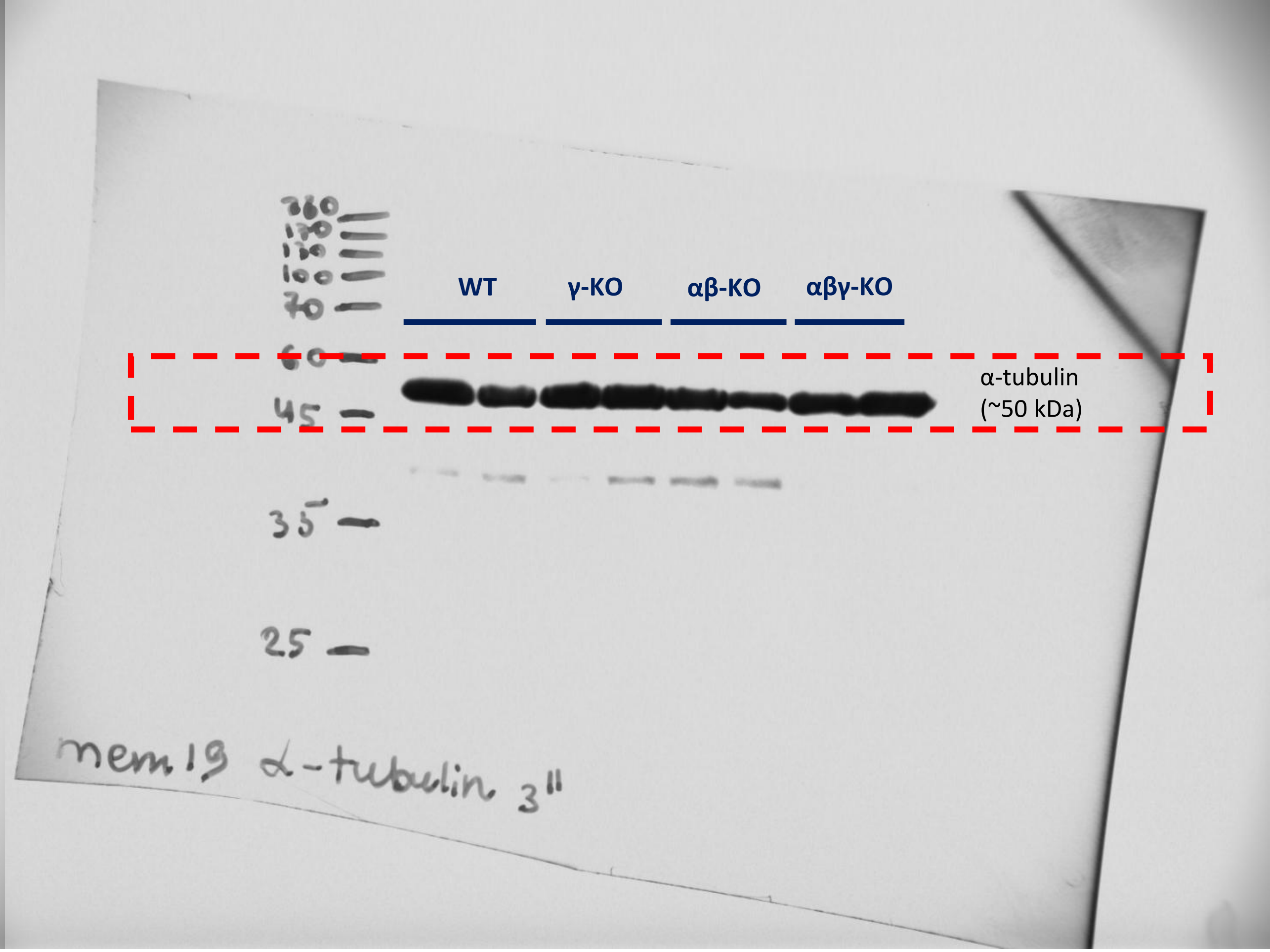
260
170
130
100
70
60
45
35
25
WT
γ-KO
αβ-KO
αβγ-KO
α-tubulin
(~50 kDa)
mem 1g α-tubulin 3''

Cortex
