# Supplementary material for: Distinct Role of γ-Synuclein in the Regulation of Motor Performance and Behavioral Responses in Mice
Source: Biomedicines. 2026 Jan 2;14(1):92. doi: 10.3390/biomedicines14010092 (PMC12839295; doi:10.3390/biomedicines14010092)
Supplement: Supplementary file 1 [file biomedicines-14-00092-s001.zip › FigureS2.pdf]

Figure S2. Representative images and uncropped Western blots for tyrosine hydroxylase in the brains of 13-month-old synuclein KO (γ-KO, αβ-KO, and αβγ-KO) and WT mice.

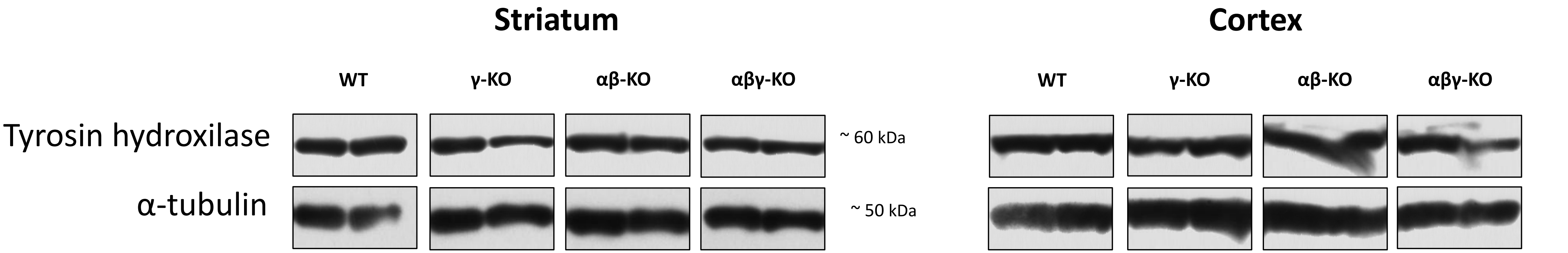

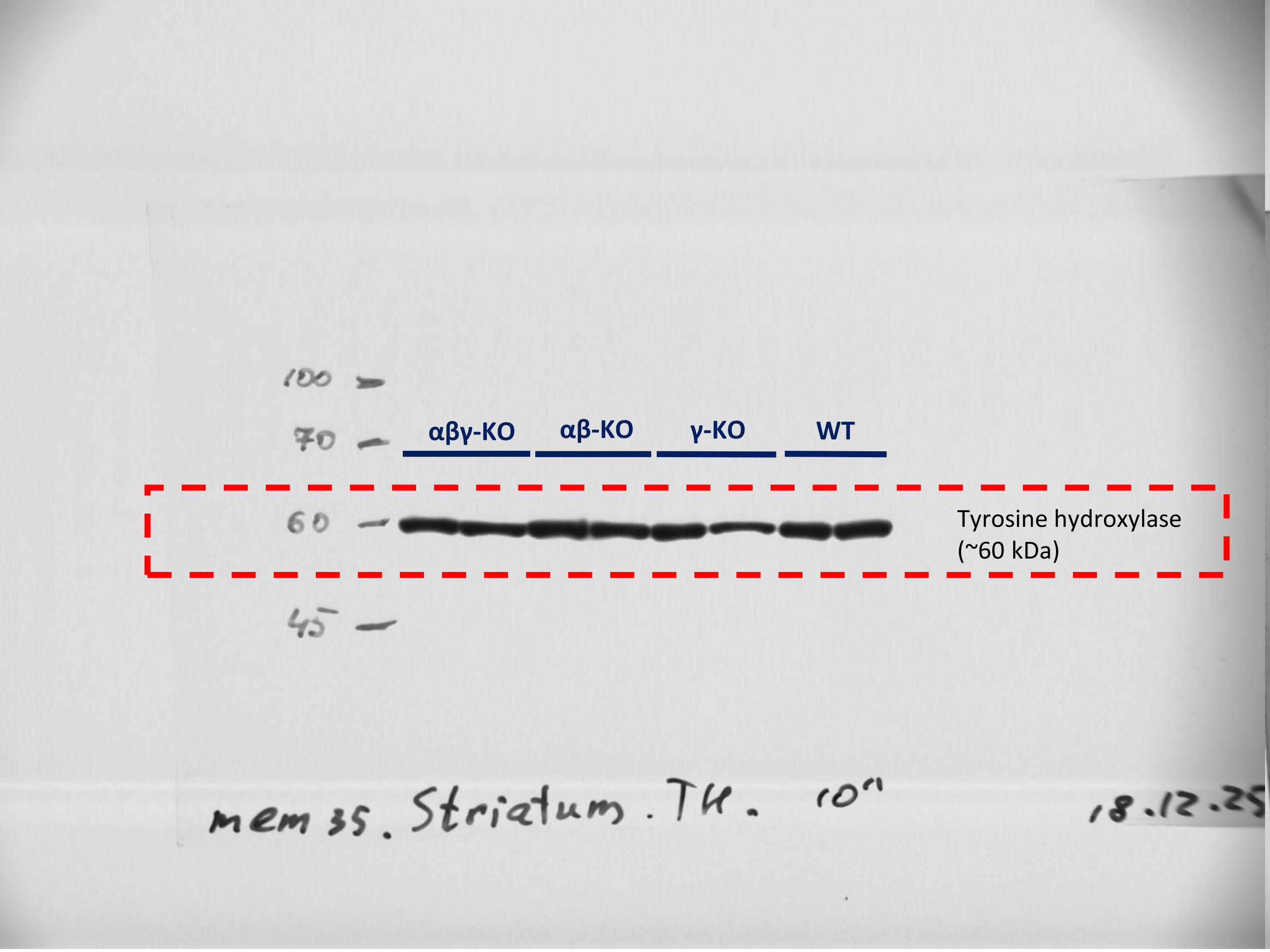
100
70
60
45
αβγ-KO
αβ-KO
γ-KO
WT
Tyrosine hydroxylase
(~60 kDa)
mem 35. Striatum. TH. 10"
18.12.25

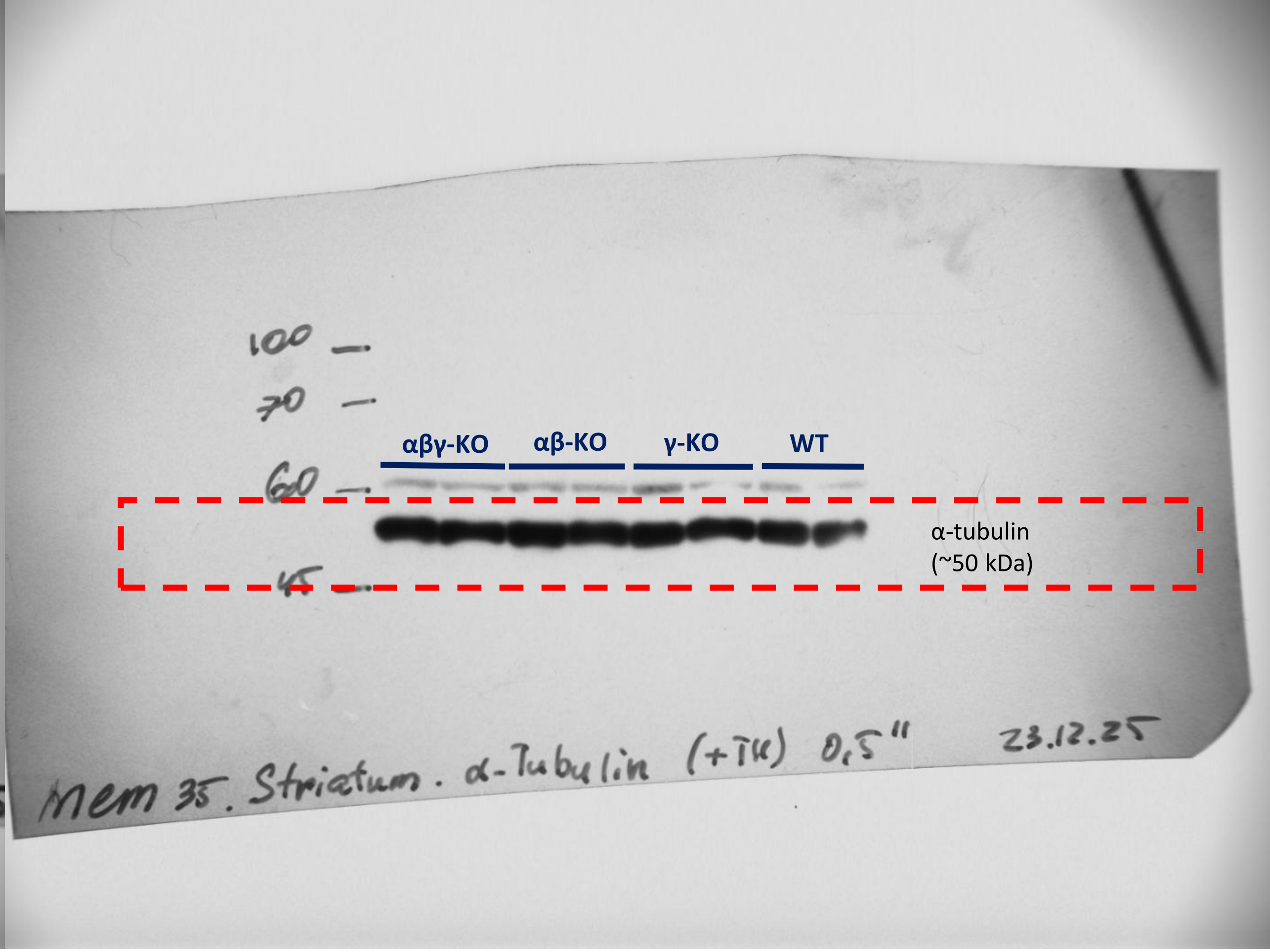
100
70
60
45
αβγ-KO
αβ-KO
γ-KO
WT
α-tubulin
(~50 kDa)
mem 35. Striatum. α-Tubulin (+TH) 0,5"
23.12.25

Striatum

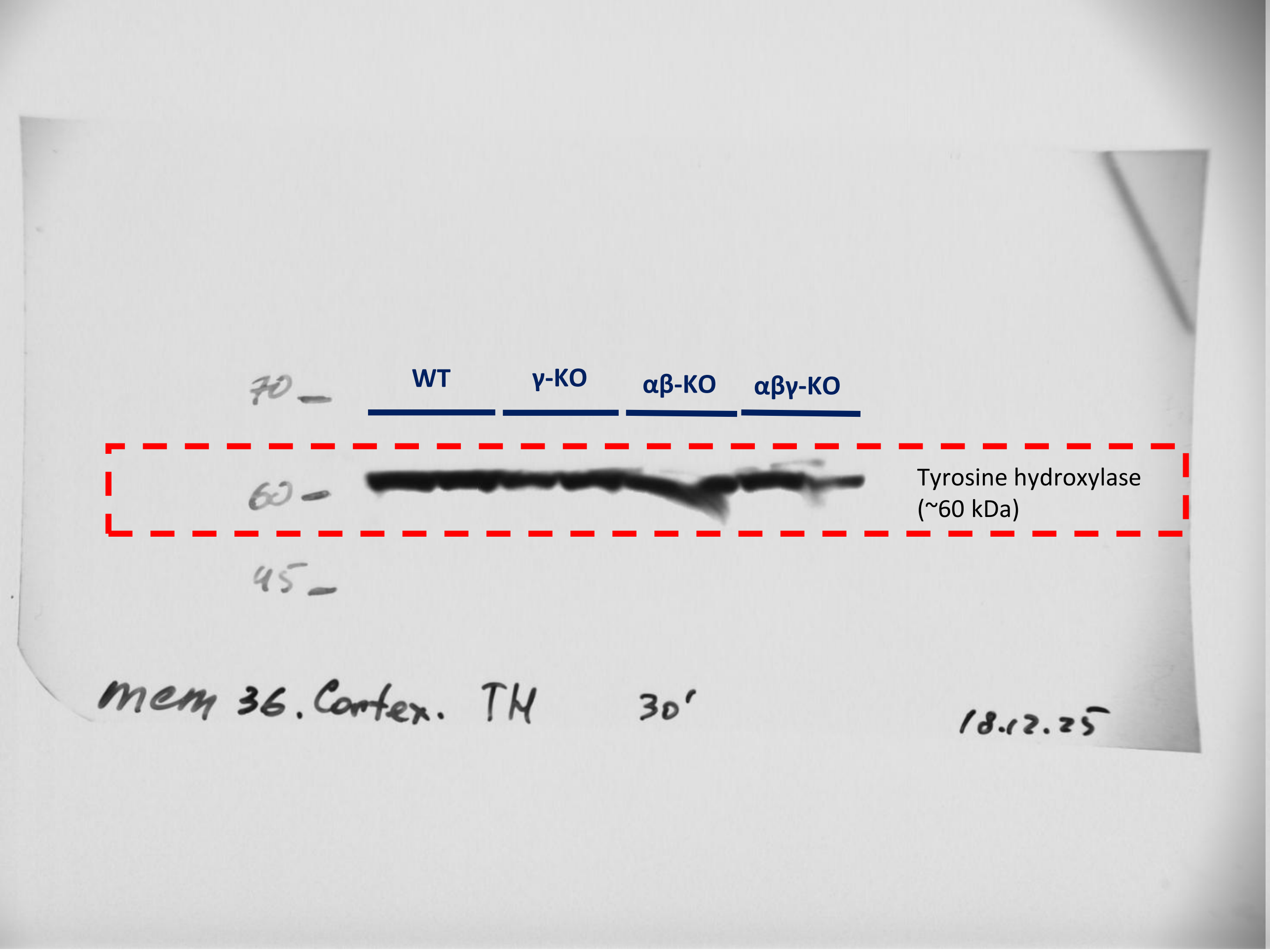
70
60
45
WT
γ-KO
αβ-KO
αβγ-KO
Tyrosine hydroxylase
(~60 kDa)
mem 36. Cortex. TH
30'
18.12.25

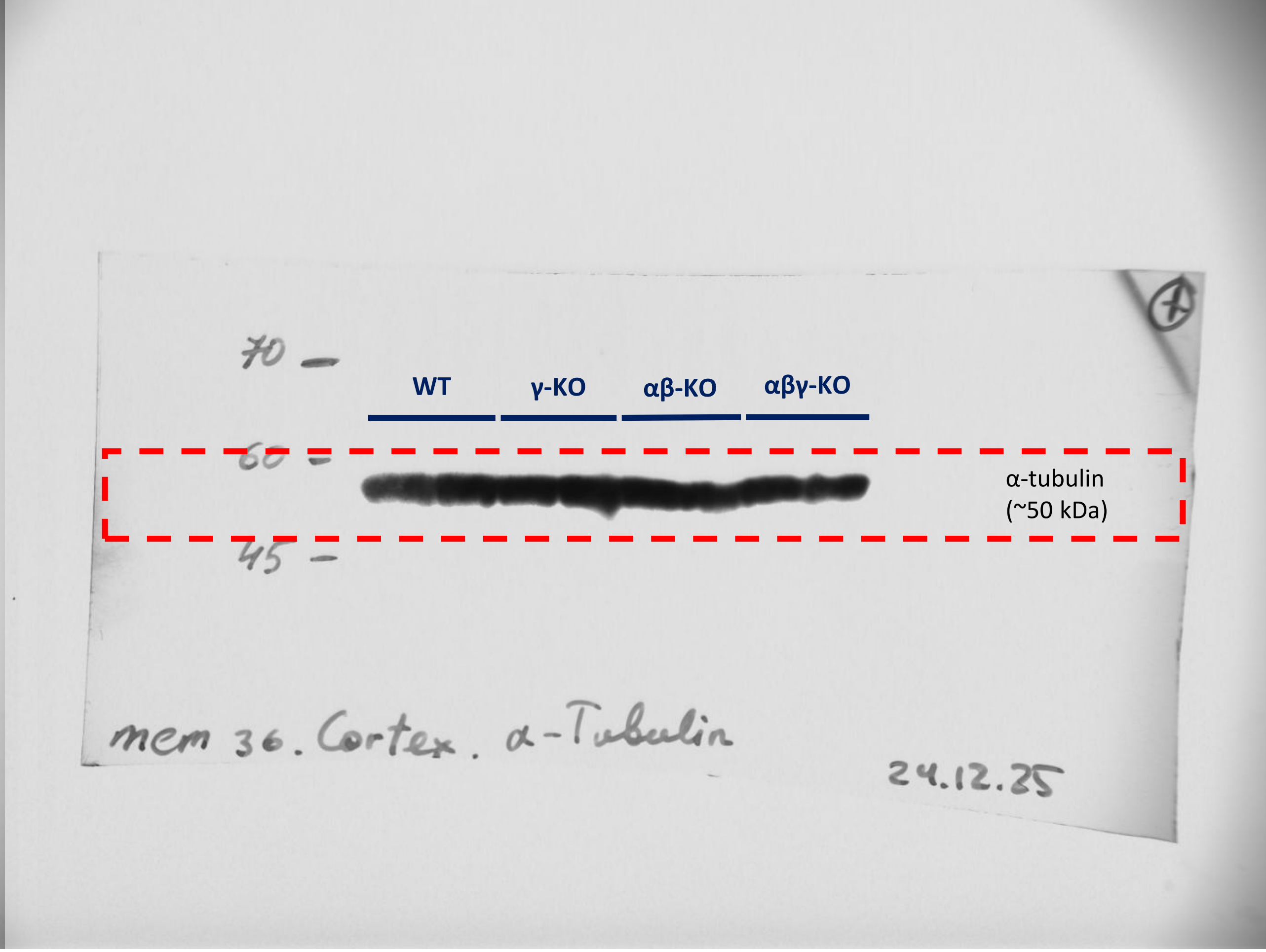
70
60
45
WT
γ-KO
αβ-KO
αβγ-KO
α-tubulin
(~50 kDa)
mem 36. Cortex. α-Tubulin
24.12.25

Cortex
